# Noise-evoked dopamine dynamics in the nucleus accumbens and tail of the striatum

**DOI:** 10.1016/j.isci.2026.115789

**Published:** 2026-04-17

**Authors:** Takafumi Furuyama, Munenori Ono, Nobuo Kato, Ryo Yamamoto

**Affiliations:** 1 Department of Physiology, Kanazawa Medical University, Uchinada, Ishikawa 920-0293, Japan

**Keywords:** molecular biology, molecular neuroscience, sensory neuroscience

## Abstract

Sound is essential for threat detection. In many animals, salient sounds trigger defensive behaviors, suggesting an innate negative valence. Here, we examined how salient noise is represented in dopaminergic circuits encoding valence and saliency. In the nucleus accumbens (NAc), dopamine decreased during noise presentation in all subjects, with a subset additionally showing a transient increase at noise onset. Dopaminergic fibers from the ventral tegmental area showed corresponding patterns in the NAc. In the tail of the striatum (TS), dopamine increased in response to noise, also suggesting negative valence coding. For ramped and damped noises, dopamine dynamics are related to the temporal profile of sound intensity. Neural activity increased in both NAc and TS during noise. Together, these findings suggest that dopaminergic signaling in the NAc and TS represents noise intensity with negative valence, while NAc dopamine also reflects perceived saliency and may contribute to defensive behaviors evoked by auditory stimuli.

## Highlights

• In the NAc, dopamine decreased during noise presentation
• A subset additionally showed a transient dopamine increase at noise onset
• In the TS, dopamine increased during noise presentation
• Neural activities in the NAc and TS increased during noise presentation

## Graphical abstract

**Figure:**
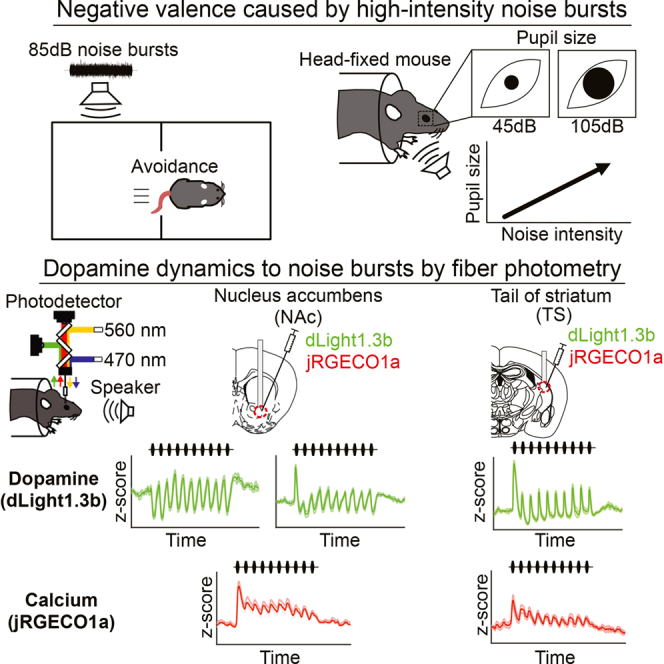

## Introduction

Avoiding potential threats is crucial for animal survival, and auditory information is an essential cue for perceiving such threats.^1^^,^^2^ Many animals avoid salient sounds, even though the sound is not associated with a specific danger.^3^^,^^4^^,^^5^^,^^6^^,^^7^ Various defensive behaviors induced by tone or noise have been reported in rodents. For instance, it is well known that tone or noise induces startle behaviors in rats and mice,^8^^,^^9^ and the startle is enhanced by stress or associative fear conditioning.^8^^,^^10^^,^^11^ The auditory looming (ramped noise) also triggers avoidance in mice, whereas damped noise causes mice to freeze.^12^ Rats avoid noise more than pure tone.^13^ Salient sound stimuli trigger flight behaviors in a fearful context in mice.^14^^,^^15^ These previous reports suggest that the salient sounds involve an innate negative valence for rodents.

In the central nervous system, dopamine neurons in the ventral tegmental area (VTA), which have dense projections to the striatum and nucleus accumbens (NAc), are regarded as one of the neural bases representing valence.^16^^,^^17^^,^^18^^,^^19^ The dopaminergic dynamics in the NAc are also considered to represent valence.^20^^,^^21^ In general, the increase of dopamine in the striatum and NAc correlates with reward, and the decrease of dopamine correlates with punishment or aversive stimuli, while various dopamine dynamics have been reported across different subregions of the NAc.^13^^,^^22^^,^^23^^,^^24^^,^^25^ In the tail of the striatum (TS), dopaminergic dynamics are reported to represent the affective aspect of stimuli distinctly from the dopaminergic system in the NAc. In this area, an increase in dopamine is correlated with aversive stimuli or predictive threats.^26^^,^^27^ Not only the direct representation of valence, but the dopaminergic dynamics in the NAc are also known to encode the incentive saliency of the stimulus^28^^,^^29^^,^^30^^,^^31^ and the perceived saliency.^32^ Thus, dopaminergic dynamics in the striatum-related region encode multiple aspects of various incoming stimuli.

Therefore, to understand the neural processing of salient noise, investigating dopamine dynamics during noise exposure is critical. Several studies have reported dopamine dynamics in the striatum and the related structures in response to noise stimuli in rats^13^^,^^25^ and mice.^33^ However, these investigations were conducted under freely moving conditions, where the animal’s head position relative to the sound speaker was variable. This limits the accuracy for assessing the quantitative relationship between sound pressure and dopaminergic responses.

Thus, the present study aimed to clarify the precise dopaminergic dynamics in the NAc and TS during noise exposure, to determine whether this system encodes negative valence, perceived saliency, or both of incoming salient sound stimuli. To achieve this, we performed fiber photometry to measure dopamine signals (dLight1.3b^34^; a dopamine sensor) and dendritic activity of medium spiny neurons (MSNs) (jRGECO1a^35^; a red-shifted calcium sensor) in response to various noise intensities from the NAc and TS of head-fixed mice. GCaMP8f, a genetically encoded calcium sensor,^36^ was also used for measuring the activities of dopaminergic fibers innervating the NAc from the VTA. Furthermore, dopaminergic responses to ramped and damped noise were investigated. Our experiment thus demonstrated that dopaminergic systems in the NAc and TS encode the information of noise stimuli in a complex manner, representing both negative valence and perceived saliency.

## Results

### The noise bursts elicited avoidance and autonomic responses

**Figure 1:**
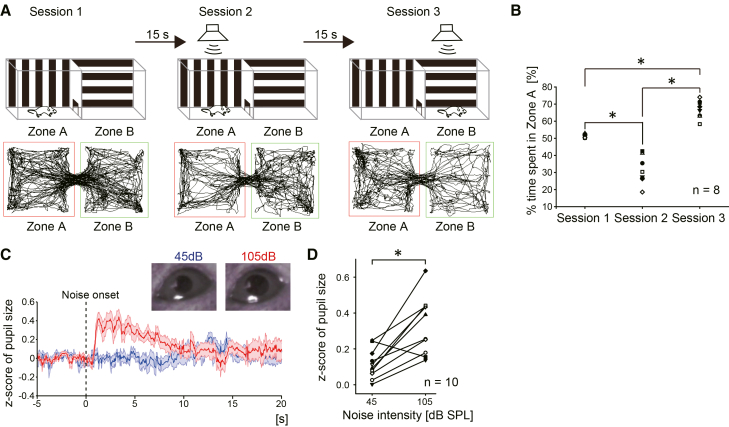
Innate behavioral and autonomic response to the loud noise burst presentations (A) Two-compartment box noise avoidance test. The schedule of this test is drawn in the upper row. The representative tracks of a subject are shown in the lower row. In session 1 (no noise presentation), the subject explored the two compartments equally. In session 2, the subject avoided the compartment (Zone A) where the noise burst was presented upon entering the area. In session 3, the compartment presenting the noise was flipped. The subject avoided the compartment (Zone B) where the noise burst was presented, the same as in session 2. (B) Summary of the time spent in Zone A during the series of sessions. The subjects avoided the compartment noise presented. Each symbol corresponds to each subject (*n* = 8). (C) The changes in pupil size during two intensities of noise were measured. *Left*: the representative *Z* score traces of pupil size during the noise presentations. The blue and red traces represent the responses during 45 and 105 dB (10 s) noise presentations, respectively. Pictures show the typical pupil size during the noise presentations. (D) The summary of pupil size during the noise presentations. The pupil size was significantly larger during the 105 dB noise presentation than during the 45 dB noise presentation. Each symbol corresponds to each subject (*n* = 10). ∗ indicates *p* < 0.05.

First, we examined the effects of loud noise burst presentations on exploring behaviors and autonomic responses of mice. The two-compartment noise avoidance test was employed to test the effects of noise on free exploration. The design of this test is shown in Figure 1A. The subjects explored two compartments almost equally in session 1 when no noise was presented (Figure 1A, left). In session 2, the subjects avoided the compartment where the noise (85 dB) was presented (Zone A; Figure 1A, middle). In session 3, the noise was presented in another compartment, Zone B. The subjects also avoided the compartment noise presented (Zone B; Figure 1A, right). The percentage of time spent in Zone A was 55.5 ± 0.3%, 31.1 ± 2.9%, and 66.9 ± 1.8% in sessions 1, 2, and 3, respectively (Figure 1B; paired *t* test followed by Bonferroni correction, df = 7, session 1 vs. session 2, t = 7.29, *p* < 0.001; session 2 vs. session 3, t = −9.75, *p* < 0.001; session 1 vs. session 3, t = −7.48, *p* < 0.001; *n* = 8). We also tested the effects of noise on autonomic responses by measuring the pupil size during the noise bursts. The pupil was dilated when the loud noise bursts (105 dB, 10 s) were presented, while the soft noise bursts (45 dB, 10 s) did not alter the pupil size (Figures 1C and 1D ; *Z* score for 45 dB, 0.12 ± 0.03; 105 dB, 0.33 ± 0.05; paired *t* test, df = 9, t = −4.40, *p* = 0.0017; *n* = 10). These results confirmed that mice avoid loud noises and exhibit autonomic responses, as indicated by pupillary dilation. These suggest that the loud noise itself has a negative valence for mice.

### Two types of dopamine dynamics during the noise bursts were confirmed

**Figure 2:**
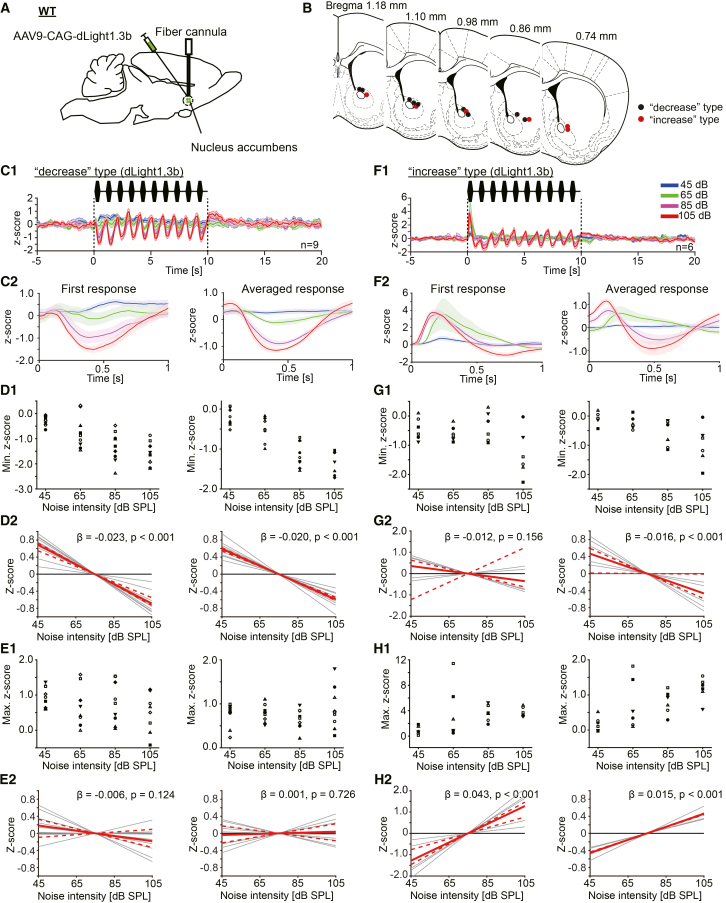
The dopamine dynamics in the nucleus accumbens (NAc) during the loud noise burst presentations. Two types of dopaminergic dynamics were observed (A) The schema indicates the recording configuration. AAV9-CAG-dLight1.3b was injected into the NAc of wild-type mice, and fiber photometry was performed from the NAc. (B) Recording sites were plotted. The red and black dots represent the recording sites exhibiting “increase” and “decrease” dopamine responses, respectively. (C1) The dopamine response of “decrease” type subjects. Average z-scores of the dopamine signal during noise burst presentations (45–105 dB) are shown (*n* = 9). As the noise intensity increased, the dopamine signal decreased prominently. (C2) *Left*: Responses correspond to the first noise burst. *Right*: The average responses for 10 noise bursts. Each color corresponds to noise intensities. (D1) *Left*: The minimum z-scores of the response during the first noise burst are plotted. *Right*: The minimum z-scores of the average response during each noise burst are plotted. (D2) *Left and right*: Linear regression lines for minimum z-scores are shown. Gray lines indicate the regression line for each subject. The red line indicates the common regression line, and the red dotted lines indicate its CI. The same conventions are used in E2, G2, and H2. A significant linear relationship between the minimum *Z* score and noise intensity was confirmed in both first and average responses for “decrease” type subjects. (E1) *Left*: The maximum z-scores of the response during the first noise burst are plotted. *Right*: The maximum z-scores of the average response during each noise burst are plotted. Each symbol corresponds to each subject. (E2) *Left and right*: No significant linear relationship between the maximum *Z* score and noise intensity was confirmed in both first and average responses for “decrease” type subjects. (F1) The dopamine response of “increase” type subjects. Average z-scores of the dopamine signal during noise burst presentations (45–105 dB) are shown (*n* = 6). (F2) *Left*: Responses correspond to the first noise burst. *Right*: The average responses for 10 noise bursts. Each color corresponds to noise intensities. (G1) *Left*: The minimum z-scores of the response during the first noise burst are plotted. *Right*: The minimum z-scores of the average responses for 10 noise bursts are plotted. (G2) *Left, first response*: No significant linear relationship between the maximum *Z* score and noise intensity was confirmed. *Right, average response*: A significant linear relationship between the minimum *Z* score and noise intensity was confirmed for “increase” type subjects. (H1) *Left*: The maximum z-scores of the response during the first noise burst are plotted. *Right*: The maximum z-scores of the average response for 10 noise bursts are plotted. Each symbol corresponds to each subject. (H2) *Left and right*: A significant linear relationship between the maximum *Z* score and noise intensity was confirmed in both first and average responses for “increase” type subjects.

To observe the dopamine dynamics during the noise bursts, dLight1.3b, a dopamine biosensor, was introduced in the NAc by AAV9-CAG-dLight1.3b injection. The fiber photometry method was applied to measure the dopamine dynamics in the NAc. The recording configuration is shown in Figure 2A. The recording sites are shown in Figure 2B. We identified two subject types based on dopamine dynamics during the noise bursts. The first of these we termed the “decrease” type. As shown in Figure 2C1, the dopamine signal decreased corresponding with each noise burst. The extent of the decrease was noise intensity dependent (Figures 2C1, 2C2, 2D1). A strong linear relationship between the extent of the first decrease and the noise intensity was confirmed (Figure 2D1 *left* and D2 *left*; wild bootstrap *t* test, β = −0.023 ± 0.003, t = −7.92, *p* < 0.001, CI = [-0.024, −0.018], *n* = 9). The extent of the average decrease depended on the noise intensity, as well (Figure 2D1 *right* and D2 *right*; wild bootstrap *t* test, β = −0.020 ± 0.002, t = −9.61, *p* < 0.001, CI = [-0.021, −0.018], *n* = 9). Additionally, a rebound-like increase of the dopamine signal followed each noise burst (Figures 2C1 and 2C2). This increase was not apparent at the first noise burst, while it became relatively apparent with the repetition of bursts. The extent of the first increase did not exhibit significant linear relationship with the noise intensity (Figure 2C2 *left*, E1 *left*, E2 *left*; wild bootstrap *t* test, β = −0.006 ± 0.003, t = −1.78, *p* = 0.124, CI = [-0.011, 0.003]; *n* = 9), also the extent of the average increase did not correlate (Figure 2C2 *right*, E1 *right*, E2 *right*; wild bootstrap *t* test, β = 0.001 ± 0.003, t = 0.40, *p* = 0.726, CI = [-0.006, 0.007]; *n* = 9). The second type is the “increase” type. As shown in Figures 2F1 and 2F2, the dopamine signal showed a steep increase at the onset of the first noise burst presentation. This steep increase was not apparent at the onset of the 2nd to 10th burst presentation. Similar to the “decrease” type response, during the 2nd to 10th noise burst presentation, the reduction of the dopamine signal, followed by a rebound-like increase, was observed. The extent of the first steep increase dependent on noise intensity (Figure 2F2 *left*, H1 *left*, H2 *left*; wild bootstrap *t* test, β = 0.043 ± 0.009, t = 4.52, *p* < 0.001, CI = [0.026, 0.049]; *n* = 6), also the extent of the averaged rebound-like increase response was noise intensity dependent (Figure 2F2 *right*, H1 *right*, H2 *right*; wild bootstrap *t* test, β = 0.015 ± 0.001, t = 10.19, *p* < 0.001, CI = [0.015, 0.016]; *n* = 6). The extent of the first decrease during each noise burst presentation did not correlate with the noise intensity (Figure 2F2 *left*, G1 *left*, G2 *left*, the first response; wild bootstrap *t* test, β = −0.012 ± 0.007, t = −1.67, *p* = 0.156, CI = [-0.021, 0.041]; *n* = 6), while the average decrease depended on the noise intensity (Figure 2F2 *right*, G1 *right*, G2 *right*, the averaged response; wild bootstrap *t* test, β = −0.016 ± 0.005, t = −3.25, *p* < 0.001, CI = [-0.020, −0.000]; *n* = 6). These results suggest that there are two types of dopaminergic dynamics in the NAc during noise presentation. Note that during the noise presentation, no obvious startle was observed in all subjects. Each value for the figures are listed in the supplementary table (Table S1).

### Except for the response to the first noise burst, the two types of dopamine dynamics share the common feature of a reduction in dopamine during the presentation of noise bursts

**Figure 3:**
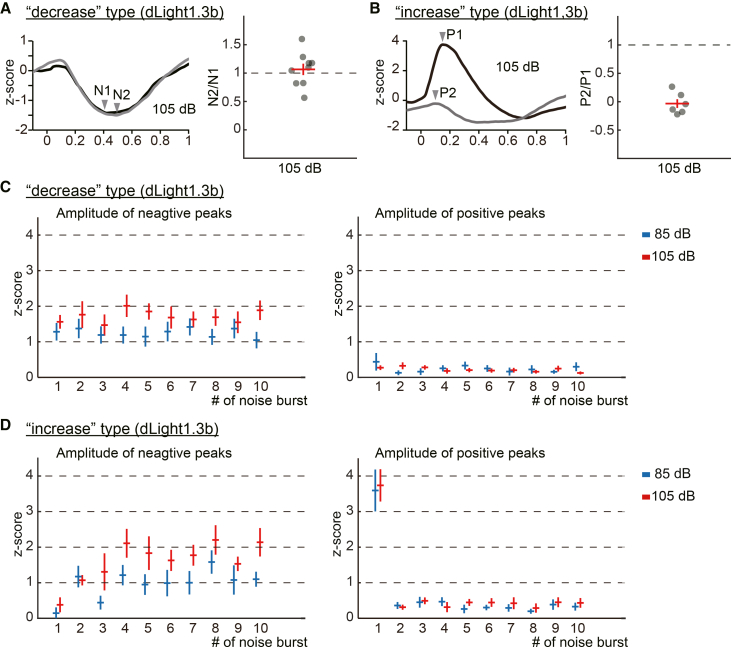
Reduction of dopamine signal is the primary feature during noise burst presentations (A) *Left*: The dopamine signals of the “decrease” type during the first and second noise bursts are superimposed. Arrowheads indicate the first negative peak (N1) and the second negative peak (N2). *Right*: Ratios of N2/N1 during 105 dB noise presentations are plotted. Red horizontal lines indicate the means, and vertical lines indicate S.E.M. The extent of signal reduction was stable over the repetitive noise presentations. (B) *Left*: The dopamine signals of the “increase” type during the first and second noise bursts are superimposed. Arrowheads indicate the first positive peak (P1) and the second positive peak (P2). *Right*: Ratios of P2/P1 during 105 dB noise presentations are plotted. Red horizontal lines indicate the means, and vertical lines indicate S.E.M. The steep increase in signal disappeared at the second noise presentation. (C) Absolute *Z* score amplitudes of negative and positive peaks during each noise burst (1st to 10th) are plotted for the “decrease” type dopamine dynamics. Each amplitude was calculated using the value at the onset of each noise burst as the baseline. Horizontal bars indicate means, and vertical bars indicate S.E.M. (blue, 85 dB; red, 105 dB). (D) Absolute *Z* score amplitudes of negative and positive peaks during each noise burst (1st to 10th) are plotted for the “increase” type dopamine dynamics. Each amplitude was calculated using the value at the onset of each noise burst as the baseline. Horizontal bars indicate means and vertical bars indicate S.E.M. (blue, 85 dB; red, 105 dB). The amplitudes of negative peaks are larger than those of positive peaks in both “decrease” and “increase” types, except for the first peaks.

Our observation of two dopamine response subject types, the “decrease” and “increase,” led us to hypothesize that dopamine reactions to the second and subsequent noise bursts are essentially uniform. We assumed that the divergence between the two subject types emerged only at the onset of noise bursts. To test this idea, we quantified and compared the dopaminergic responses to the first-burst and second-burst for each type. As shown in Figure 3A (left traces), the amplitude of the first negative peak (N1) and the second negative peak (N2) of the “decrease” type were almost the same at the noise intensities of 105 dB. The ratio of N2/N1 was 1.07 ± 0.10 for 105 dB (Figure 3A *right*, *n* = 9). In the case of the “increase” type, the steep first positive peak (P1) was induced by the first noise burst, while the second positive peak (P2) to the second noise burst was not apparent at the noise intensities of 105 dB, as shown in Figure 3B (left traces). The ratio of P2/P1 was −0.03 ± 0.08 for 105 dB (Figure 3B *right*, *n* = 6). These results suggest that repeated noise burst presentations stably induce a decrease in dopamine in response to the noise burst. In contrast, the steep increase in dopamine is not reproduced by repeated noise burst presentations. These also support the idea that the difference between the two types of dopamine dynamics is whether or not the initial steep dopamine increase is present. To further confirm this idea, we examined the positive and negative peak amplitudes of dopamine dynamics during noise burst presentations. Each absolute amplitude was calculated using the value at the onset of each noise burst as the baseline. The *Z* score amplitudes of the negative peaks in the “decrease” type remained consistently in the range of one to two across repeated noise burst presentations (Figure 3C; *left*). In contrast, the *Z* score amplitudes of the positive peaks were consistently lower than those of the negative peaks (Figure 3C; *right*). These findings indicate that a reduction in dopamine signal is a consistent characteristic of the “decrease” type dynamics during loud noise presentations. In the case of the “increase” type dynamics, the *Z* score amplitude of the first positive peak was markedly larger than that of the subsequent peaks (Figure 3D *right*). For 85 dB noise bursts, the amplitude of the first peak was 3.59 ± 0.59, while the average amplitude of the subsequent peaks was 0.34 ± 0.04. A similar pattern was observed for 105 dB bursts (the first peak, 3.74 ± 0.45; average of the subsequent peaks, 0.40 ± 0.11). The amplitudes of subsequent positive peaks were comparable to those observed in the “decrease” type. The *Z* score amplitudes of the negative peaks were lower in the first several responses than in the remaining ones (Figure 3D *left*), since the first steep increase of signal overrides the decrease response. With the influence of the first positive peak declining, the amplitude of negative peaks increased and became stable between one to two. After the negative peaks became stable, the amplitudes of negative peaks were consistently larger than those of positive peaks. These results support the idea that noise bursts reduce the dopamine signal in both “decrease” and “increase” subject types, except for the first response. Taken together, these results may suggest that dopamine dynamics in the NAc generally encode intense noise as a stimulus with negative valence. In addition, a transient increase in dopamine was observed at the onset of noise bursts in a subset of subjects, suggesting that NAc dopamine also encodes perceived saliency, particularly at onset. Each value for the figures is listed in the supplementary table (Table S2).

### Two types of VTA fiber activities in the NAc during the noise burst presentations were confirmed

**Figure 4:**
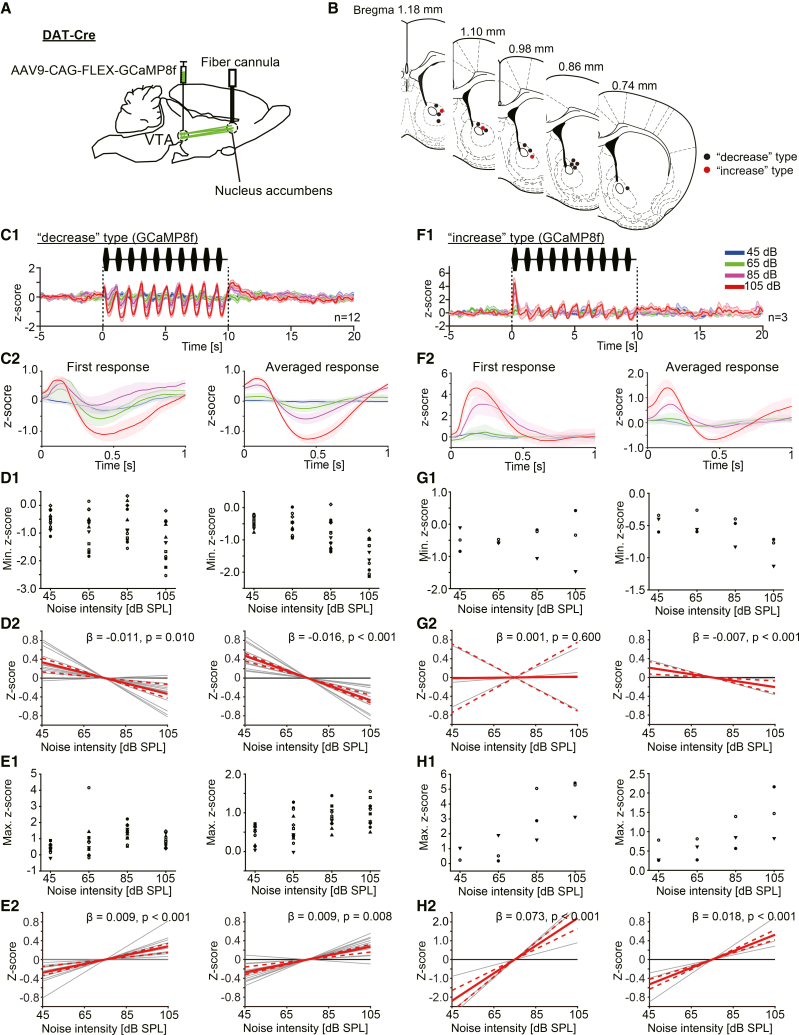
The activities of VTA fibers in the NAc during the loud noise burst presentations. Two types of activity changes were observed (A) The schema indicates the recording configuration. AAV9-CAG-FLEX-GCaMP8f was injected into the VTA of DAT-Cre mice, and fiber photometry was performed from the NAc. (B) Recoding sites were plotted. The red and black dots represent the recording sites exhibiting “increase” and “decrease” of VTA fiber activities, respectively. (C1) The response of “decrease” type subjects. Average z-scores of the VTA fiber activity during noise burst presentations (45–105 dB) are shown (*n* = 12). As the noise intensity increased, the VTA activity decreased prominently. (C2) *Left*: Responses correspond to the first noise burst. *Right*: The average responses for 10 noise bursts. Each color corresponds to noise intensities. (D1) *Left*: The minimum z-scores of the response during the first noise burst are plotted. *Right*: The minimum z-scores of the average response during each noise burst are plotted. (D2) *Left and right*: Linear regression lines for minimum z-scores are shown. Gray lines indicate the regression line for each subject. The red line indicates the common regression line, and the red dotted lines indicate its CI. The same conventions are used in E2, G2, and H2. A significant linear relationship between the minimum *Z* score and noise intensity was confirmed in both first and average responses for “decrease” type subjects. (E1) *Left*: The maximum z-scores of the response during the first noise burst are plotted. *Right*: The maximum z-scores of the average response during each noise burst are plotted. Each symbol corresponds to each subject. (E2) *Left and right*: A significant linear relationship between the maximum *Z* score and noise intensity was confirmed in both first and average responses for “decrease” type subjects. (F1) The response of “increase” type subjects. Average z-scores of the VTA fiber activity during noise burst presentations (45–105 dB) are shown (*n* = 3). (F2) *Left*: Responses correspond to the first noise burst. *Right*: The average responses for 10 noise bursts. Each color corresponds to noise intensities. (G1) *Left*: The minimum z-scores of the response during the first noise burst are plotted. *Right*: The minimum z-scores of the average responses for 10 noise bursts are plotted. (G2) *Left, first response*: No significant linear relationship between the maximum *Z* score and noise intensity was confirmed. *Right, average response*: A significant linear relationship between the minimum *Z* score and noise intensity was confirmed for “increase” type subjects. (H1) *Left*: The maximum z-scores of the response during the first noise burst are plotted. *Right*: The maximum z-scores of the average response for 10 noise bursts are plotted. Each symbol corresponds to each subject. (H2) *Left and right*: A significant linear relationship between the maximum *Z* score and noise intensity was confirmed in both first and average responses for “increase” type subjects.

Given that we confirmed two types of dopamine dynamics during noise presentations in the NAc, a question arises whether the dopamine fibers projecting from the VTA to the NAc also exhibit these two types of activity. To observe the dopamine fiber activities in the NAc, GCaMP8f, a calcium biosensor, was introduced in the VTA by AAV9-CAG-FLEX-GCaMP8f injection into DAT-Cre mice. The fiber photometry technique was used to measure dopamine fiber activity in the NAc. The recording configuration is shown in Figure 4A. The recording sites are shown in Figure 4B. Two subject types of VTA dopamine fiber activities, similar to the two types of dopamine dynamics, were also observed during the noise burst presentations. Same as the dopamine dynamics, we defined two subject types of activities as the “decrease” and “increase” subject types. The “decrease” type fiber activity is shown in Figure 4C1. The VTA fiber activities decreased corresponding with each noise burst. The extent of the decrease was noise intensity dependent (Figures 4C1, C2, D1). A linear relationship between the extent of the first decrease and the noise intensity was confirmed (Figure 4D2 *left*; wild bootstrap *t* test, β = −0.011 ± 0.003, t = −3.96, *p* = 0.010, CI = [-0.014, −0.004]; *n* = 12). The extent of the average decrease depended on the noise intensity, as well (Figure 4D2 *right*; wild bootstrap *t* test, β = −0.016 ± 0.002, t = −6.40, *p* < 0.001, CI = [-0.018, −0.012]; *n* = 12). Also, the rebound-like increases of the dopamine signal followed each noise burst (Figure 4C1 and C2). The extent of the first and averaged increase exhibited a linear relationship with the noise intensity (Figure 4C2 *left*, E1 *left*, E2 *left*, the first response, wild bootstrap *t* test, β = 0.009 ± 0.002, t = 4.46, *p* < 0.001, CI = [0.005, 0.011]; E1 *right* and E2 *right*, the average response, wild bootstrap *t* test, β = 0.009 ± 0.002, t = 4.80, *p* = 0.008, CI = [0.005, 0.011]; *n* = 12). The second subject type, “increase” type, of fiber activity is shown in Figure 4F1. The fiber activity showed a steep increase at the onset of the first noise burst presentation, the same as the “increase” type of dopamine dynamics. The characteristics of this type of activity (Figure 4F1 and F2) seem quite similar to the “increase” type of dopamine dynamics. The extent of the first and averaged increase exhibited a linear relationship with the noise intensity (Figure 4F2 *left*, H1 *left*, H2 *left*, the first response, wild bootstrap *t* test, β = 0.073 ± 0.002, t = 3.36, *p* < 0.001, CI = [0.055, 0.091]; F2 *right*, H1 *right*, H2 *right*, the average response, wild bootstrap *t* test, β = 0.018 ± 0.006, t = 2.79, *p* < 0.001, CI = [0.014, 0.021]; *n* = 3). The extent of the averaged decrease exhibited a linear relationship with the noise intensity (Figure 4F2 *right*, G1 *right*, G2 *right*, the average response, wild bootstrap *t* test, β = −0.007 ± 0.003, t = −2.11, *p* < 0.001, CI = [-0.011, −0.002]; *n* = 3), while the first decrease did not correlate with the noise intensity (Figure 4F2 *left*, G1 *left*, G2 *left*, the first response, wild bootstrap *t* test, β = 0.001 ± 0.013, t = 0.04, *p* = 0.600, CI = [-0.024, 0.025]; *n* = 3). These results suggest that the dopamine dynamics in the NAc reflect the activities of dopamine fibers from the VTA. Each value for the figures is listed in the supplementary table (Table S3).

### The activity of NAc neurons increased during the noise bursts, with dopamine decreases

**Figure 5:**
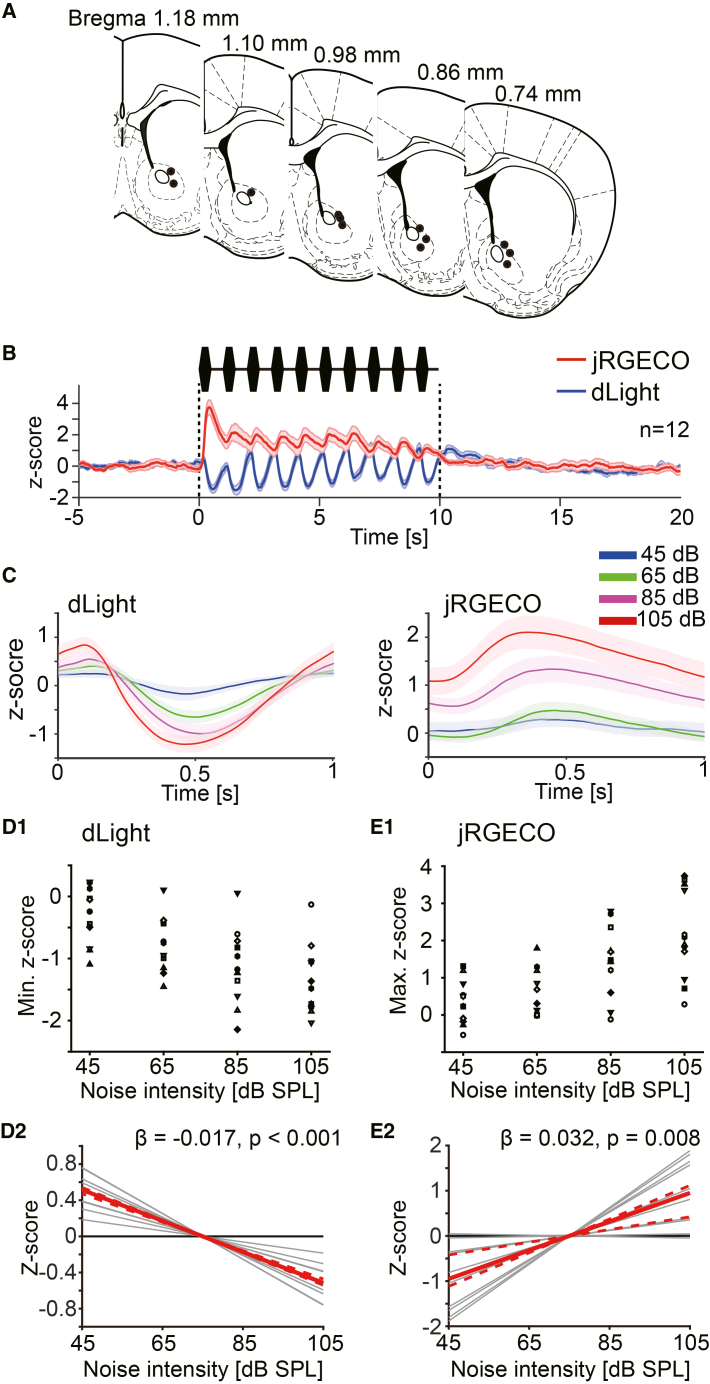
Simultaneous measurement of dopamine dynamics and neural activity in the NAc during the loud noise burst presentations (A) Recoding sites were plotted. (B) Average z-scores of dopamine signal and neural activity during noise burst presentations (105 dB) are shown (*n* = 12). (C) *Left*: The average dopamine response for 10 noise bursts. *Right*: The average neural activities for 10 noise bursts. Each color corresponds to noise intensities. (D1) The minimum z-scores of the average dopamine response for 10 noise bursts are plotted. Each symbol corresponds to each subject. (D2) A significant linear relationship between the minimum *Z* score and noise intensity was confirmed. (E1) The maximum z-scores of the average neural activity for 10 noise bursts are plotted. (E2) A significant linear relationship between the maximum *Z* score and noise intensity was confirmed.

Next, we simultaneously measured the neural activity of the NAc and the dopamine dynamics in the NAc, using jRGECO1a (a red-shifted genetically encoded calcium sensor) and dLight1.3b. The recording sites are shown in Figure 5A. In this experiment, all subjects exhibited the “decrease” type. Corresponding to each noise burst, the dopamine signal decreased, and the neural activity increased in the NAc, as shown in Figures 5B and 5C. The extent of decrease of average dopamine responses to noise bursts exhibited a linear relationship with the noise intensity (Figure 5D1 and D2; wild bootstrap *t* test, β = −0.017 ± 0.002, t = −10.15, *p* < 0.001, CI = [-0.018, −0.016]; *n* = 12). The extent of increase in neural activity also exhibited a linear relationship with the noise intensity (Figure 5E1 and E2; wild bootstrap *t* test, β = 0.032 ± 0.006, t = 4.89, *p* = 0.008, CI = [0.014, 0.037]; *n* = 12). These results may suggest that with the dopamine decrease, the activity balance between D1 receptor-expressing medium spiny neurons (D1-MSNs) and D2 receptor-expressing MSNs (D2-MSNs) could be altered by noise stimulation, depending on its intensity. Each value for the figures is listed in the supplementary table (Table S4).

### Dopamine increased in the TS during the noise bursts

**Figure 6:**
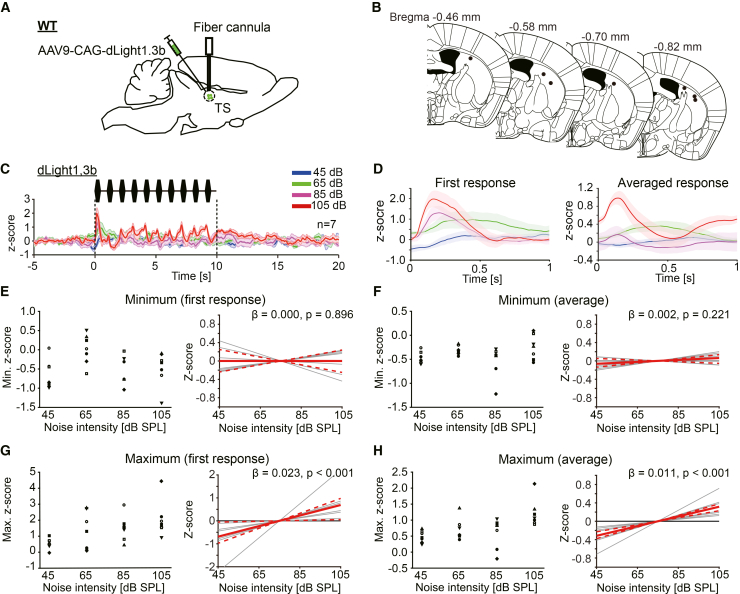
The dopamine dynamics in the tail of the striatum (TS) during the loud noise burst presentations. The dopamine signal showed a phasic increase at the presentation of the noise burst (A) The schema indicates the recording configuration. AAV9-CAG-dLight1.3b was injected into the TS of wild-type mice, and fiber photometry was performed from the TS. (B) Recoding sites were plotted. (C) Averaged z-scores of the dopamine signal during noise burst presentations (45–105 dB) are shown (*n* = 7). As the noise intensity increased, the dopamine signal increased. (D) *Left*: Responses correspond to the first noise burst. *Right*: The average responses for 10 noise bursts. Each color corresponds to noise intensities. (E) *Left*: The minimum z-scores of the response during the first noise burst are plotted. Each symbol corresponds to each subject. *Right*: Linear regression lines for minimum z-scores are shown. Gray lines indicate the regression line for each subject. The red line indicates the common regression line, and the red dotted lines indicate its CI. The same conventions are used in F, G, and H. No significant linear relationship was confirmed between the *Z* score and noise intensity. (F) *Left*: The minimum z-scores of average responses during each noise burst are plotted. *Right*: Also, no significant linear relationship was confirmed. (G) *Left*: The maximum z-scores of the response during the first noise burst are plotted. *Right*: A significant linear relationship between the maximum *Z* score and noise intensity was confirmed. (H) *Left*: The maximum z-scores of average responses during each noise burst are plotted. *Right*: Also, a significant linear relationship was confirmed.

Based on the previous reports that the reduction of dopamine in the NAc represents negative valence,^20^^,^^21^^,^^24^^,^^26^^,^^27^^,^^37^ our results thus may suggest that dopamine dynamics encode loud noise as a stimulus with negative valence. To support this idea from a different perspective, we examined dopamine dynamics in the TS, which is known to represent negative valence through dopamine increases,^26^^,^^27^ during the presentations of noise bursts. To observe the dopamine signal, dLight1.3b was introduced in the TS by AAV9-CAG-dLight1.3b injection. The fiber photometry technique was applied to measure the dopamine dynamics in the TS. The recording configuration is shown in Figure 6A. The recording sites are shown in Figure 6B. The dopamine signal in the TS exhibited a steep increase responding to the first noise burst and increased modestly during subsequent noise bursts (Figure 6C). The extent of the first steep increase was noise intensity dependent (Figure 6D *left*, 6G *left*). The extent of average increase response also depended on the intensity of the noise burst (Figure 6D *right*, 6H *left*). In both cases, statistically significant liner relationship between the extent of signal increase and the noise intensity were confirmed (Figure 6G *right*, the first increase response, wild bootstrap *t* test, β = 0.023 ± 0.009, t = 2.45, *p* < 0.001, CI = [0.002, 0.033]; 6H *right*, average increase response, wild bootstrap *t* test, β = 0.011 ± 0.003, t = 4.01, *p* < 0.001, CI = [0.007, 0.013]; *n* = 7). Different from the dopamine dynamics in the NAc, apparent reduction of dopamine signal during noise bursts was not observed (Figures 6C–6F), and there was no liner relationship between the extent of decrease and intensity of the noise (Figure 6E right, the first decrease response, wild bootstrap *t* test, β = 0.000 ± 0.003, t = 0.00, *p* = 0.896, CI = [-0.008, 0.008]; 6F right, average decrease response, wild bootstrap *t* test, β = 0.002 ± 0.002, t = 1.36, *p* = 0.221, CI = [-0.002, 0.005]; *n* = 7). Given that it was reported that dopamine increased in the TS during aversive experiences or avoidance learning, our findings support the possibility that dopamine dynamics represent loud noise as a stimulus with negative valence. This is consistent with our present results in the NAc. Each value for the figures is listed in the supplementary table (Table S5).

### The activity of TS neurons increased during the noise bursts, with dopamine increases

**Figure 7:**
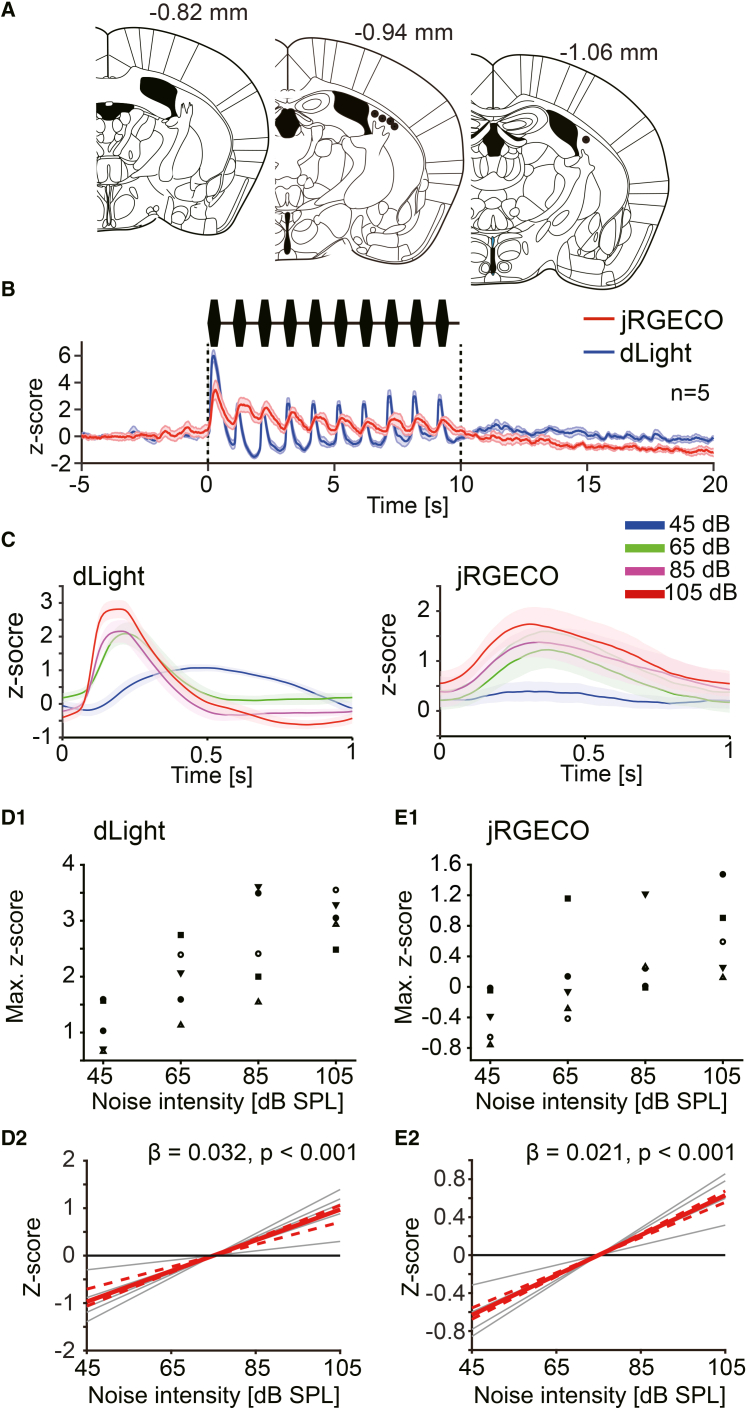
Simultaneous measurement of dopamine dynamics and neural activity in the TS during the loud noise burst presentations (A) Recoding sites were plotted. (B) Average z-scores of dopamine signal and neural activity during noise burst presentations (105 dB) are shown (*n* = 5). (C) *Left*: The average dopamine response for 10 noise bursts. *Right*: The average neural activities for 10 noise bursts. Each color corresponds to noise intensities. (D1) The maximum z-scores of the average dopamine response for 10 noise bursts are plotted. (D2) Linear regression lines for minimum z-scores are shown. Gray lines indicate the regression line for each subject. The red line indicates the common regression line, and the red dotted lines indicate its CI. A significant linear relationship was confirmed between the *Z* score and noise intensity. (E1) The maximum z-scores of the average neural activity for 10 noise bursts are plotted. (E2) A significant linear relationship was confirmed between the *Z* score and noise intensity.

Next, we simultaneously measured the neural activity of the TS and the dopamine dynamics in the TS, using jRGECO1a (a red-shifted genetically encoded calcium sensor) and dLight1.3b. The recording sites are shown in Figure 7A. Corresponding to each noise burst, both the dopamine signal and neural activity increased in the TS, as shown in Figures 7B and 7C . The extent of increase of average dopamine responses to noise bursts exhibited a linear relationship with the noise intensity (Figure 7D1 and D2; wild bootstrap *t* test, β = 0.032 ± 0.006, t = 5.19, *p* < 0.001, CI = [0.024, 0.035]; *n* = 5). The extent of increase in neural activity also exhibited a linear relationship with noise intensity (Figure 7E1 and E2; wild bootstrap *t* test, β = 0.021 ± 0.003, t = 6.74, *p* < 0.001, CI = [0.019, 0.023]; *n* = 5). These results may suggest that with the dopamine increase, the activity balance between D1-MSNs and D2-MSNs could be altered by noise stimulation, depending on its intensity, in a distinct way from the NAc. Each value for the figures is listed in the supplementary table (Table S6).

### Dopamine dynamics during ramped and damped noise presentations

**Figure 8:**
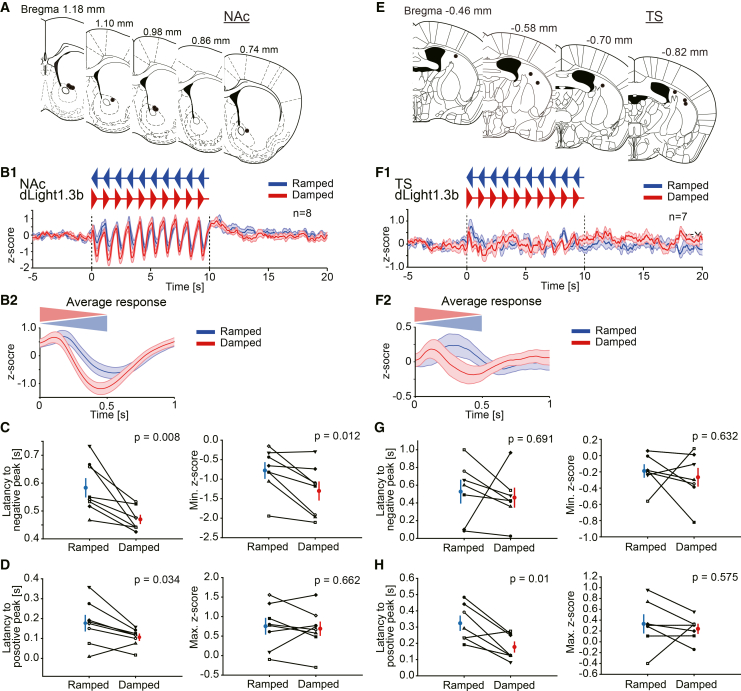
The dopamine dynamic responding to the ramped and damped noise presentations in the NAc and the TS (A) Recoding sites were plotted. (B1) Averaged z-scores of the dopamine signal in the NAc during ramped (blue) and damped (red) noise presentations (85 dB at the peak) are shown (*n* = 8). Following a brief phasic increase, the dopamine signal exhibited a decrease with distinct temporal profiles in response to ramped and damped noise stimuli. Blue and red triangles above the traces indicate the timing of noise presentations. (B2) The average responses for 10 ramped (blue) and damped (red) noises in the NAc. (C) *Left*: Latencies to the negative peak of the average trace are plotted. The latencies are longer in the ramped noise presentations than in the damped noise presentations. *Right*: The minimum z-scores of the response during the noise presentations are plotted. The magnitude of the dopamine signal decrease was significantly greater during damped noise presentations compared to ramped noise presentations. (D) *Left*: Latencies to the positive peak of the average trace are plotted. The latencies are longer in the ramped noise presentations than in the damped noise presentations. *Right*: The maximum z-scores of the response during the noise presentations are plotted. There was no statistical difference in the extent of increase between the response during ramped and damped noise presentations. (E) Recoding sites were plotted. (F1) Averaged z-scores of the photometry dopamine signal in the TS during ramped (blue) and damped (red) noise presentations (85 dB at the peak) are shown (*n* = 7). The dopamine signal exhibited an increase with distinct temporal profiles in response to ramped and damped noise stimuli. Blue and red triangles above the traces indicate the timing of noise presentations. (F2) The average responses for 10 ramped (blue) and damped (red) noises in the TS. (G) *Left*: Latencies to the negative peak of the average trace are plotted. There was no statistically significant difference in the latencies to the negative peaks between the ramped and damped noise presentations. *Right*: The minimum z-scores of the response during the noise presentations are plotted. There was no statistical difference between the ramped and damped noise presentations. (H) *Left*: Latencies to the positive peak of the average trace are plotted. The latencies are longer in the ramped noise presentations than in the damped noise presentations. *Right*: The maximum z-scores of the response during the noise presentations are plotted. There was no statistical difference in the extent of increase between the responses during ramped and damped noise presentations. Blue and red dots indicate the averages. Blue and red vertical bars indicate the S.E.M.

We have demonstrated that dopamine dynamics had a correlation with the intensity of noise. Next, we investigated whether dopamine dynamics in the NAc and TS differentially encode ramped (slow rise, fast decay) and damped (fast rise, slow decay) noises, given that a previous study reported that these auditory stimuli elicit distinct defensive behaviors.^12^ Dopamine dynamics in the NAc were measured using the same viral injection and recording configuration as illustrated in Figure 2A. The recording sites are shown in Figure 8A. In these recordings, no “increase” type subject was observed. Average *Z* score traces during noise presentations are shown in Figures 8B1 and 8B2. In response to both ramped and damp noise presentations (85 dB at the peak), the dopamine signal exhibited a transient small increase followed by a pronounced decrease. The time course of the dopamine signal generally followed the temporal profiles of the noise, ramped or damped (Figures 8B1 and 8B2). The dopamine signal decreased slowly in the case of ramped noise presented than in the case of damped noise presented (Figure 8C *left*; ramped, 0.58 ± 0.03 s; damped, 0.47 ± 0.01 s; paired *t* test, df = 7, t = 3.71, *p* = 0.008; *n* = 8). The extent of decrease was significantly greater during damped noise than ramped noise (Figure 8C *right*; ramped, −0.77 ± 0.20; damped, −1.30 ± 0.23; paired *t* test, df = 7, t = 3.38 *p* = 0.012; *n* = 8). The latency to the positive peak was also longer when the ramped noise was presented than when the damped noise was presented (Figure 8D *left*; ramped, 0.18 ± 0.04 s; damped, 0.11 ± 0.02 s; paired *t* test, df = 7, t = 2.64, *p* = 0.034; *n* = 8). There was no significant difference in the extent of increase between ramped and damped noise (Figure 8D right; ramped, 0.75 ± 0.20; damped, 0.69 ± 0.18; paired *t* test, df = 7, t = 0.46, *p* = 0.662; *n* = 8).

Next, dopamine dynamics in the TS were measured. The recording sites are shown in Figure 8E. Average *Z* score traces during noise presentations are shown in Figures 8F1 and 8F2. In response to both ramped and damped noise presentations (85 dB at the peak), the dopamine signal exhibited a phasic increase. The time course of dopamine signal change generally followed the temporal features of the noise stimulation, ramped or damped (Figures 8F1 and 8F2). The dopamine signal increased slowly in the case of ramped noise presented than in the case of damped noise presented (Figure 8H *left*; ramped, 0.32 ± 0.04 s; damped, 0.18 ± 0.03 s; paired *t* test, df = 6, t = 3.73, *p* = 0.01; *n* = 7). There was no significant difference in the extent of increase (Figure 8H *right*; ramped, 0.33 ± 0.17; damped, 0.24 ± 0.08; paired *t* test, df = 6, t = 0.59, *p* = 0.575; *n* = 7), in the latencies to negative peaks (Figure 8G *left*; ramped, 0.53 ± 0.13 s; damped, 0.46 ± 0.11 s; paired *t* test, df = 6, t = 0.42, *p* = 0.691; *n* = 7), and the extent of decrease between ramped and damped noise (Figure 8G *right*; ramped, −0.20 ± 0.08; damped, −0.30 ± 0.12; paired *t* test, df = 6, t = 0.50, *p* = 0.632; *n* = 7). Overall, dopamine dynamics during noise presentations seemed to have a correlation with the temporal profiles of intensity changes in the NAc and TS.

## Discussion

In this study, we confirmed complex dopaminergic dynamics in the NAc and TS during noise exposure. We observed two VTA-derived response patterns in the NAc: a “decrease” type, with a reduction in dopamine corresponding to noise bursts, and an “increase” type, showing a steep rise at the onset of the first noise burst. In contrast, the dopamine signal in the TS increased during noise bursts. We further confirmed that these dopamine dynamics encode noise intensity using damped and ramped noises. These results may suggest that dopamine dynamics in the NAc and TS primarily encode the negative valence of noise based on its intensity, while the NAc response may also encode perceived saliency. These dynamics likely contribute to the neural basis for defensive behaviors induced by salient auditory stimuli in rodents.

Accumulating reports suggest that the dopaminergic system in the striatum-related region, including the NAc and TS, encodes negative valence as well as positive valence.^20^^,^^21^^,^^24^^,^^26^^,^^27^^,^^37^ In general, dopamine levels in the NAc increase in response to rewards, positive experiences, or reward prediction.^13^^,^^23^^,^^24^^,^^38^^,^^39^ In contrast, a decrease in dopamine in the NAc, particularly in the core and lateral regions, was observed during aversive stimuli or negative experiences such as punishment.^13^^,^^23^^,^^24^ Our recordings were primarily conducted from the core of NAc. The dopamine reduction we observed in response to noise presentation is consistent with previous findings, suggesting that dopaminergic activity in the NAc encodes loud noise as a negatively valenced stimulus. We confirmed that the extent of dopamine decrease positively correlated with the intensity of the noise burst. Furthermore, the temporal profile of the dopamine signal reflected the envelope of ramped or damped noise. In the TS, which is thought to encode negative affective information, dopamine elevations have been reported in response to aversive stimuli.^26^^,^^27^ We also confirmed that the extent of dopamine increase in the TS during noise presentation positively correlated with the noise intensities. Thus, the intensity of noise appears to be the primary factor that causes dopamine dynamics in the NAc and TS. Some previous studies have reported dopamine increases in the ventromedial shell of the NAc even in response to negatively valenced stimuli.^22^^,^^23^^,^^40^^,^^41^ Our data also showed a modest increase in dopamine at the onset of noise in the NAc. This increase might reflect the dopaminergic transmission in the ventromedial shell, given the relatively large diameter of our photometry cannula.

In a previous study, Goedhoop and colleagues reported that the dopamine signal in the NAc also encodes the duration of noise as an aversive stimulus.^13^ However, in our present study, dopamine did not robustly represent the duration of the noise burst; rather, the signal began to return to baseline before the noise presentation had terminated, suggesting that dopamine dynamics did not represent the duration of the stimulus but rather related to its intensity. Several possible explanations may account for this discrepancy. First, the duration of noise was different between the present study (500 ms, repeated 10 times) and the previous study (5 s, continuous). This would affect the perception of the emotional aspect of auditory stimuli. Additionally, the shorter duration of noise would be difficult to accurately represent. Second, the most notable difference is that the previous study recorded from freely moving rats, whereas our recordings were conducted in head-fixed mice. In the freely moving condition, the perceived intensity of the noise may vary as the animal moves relative to the sound source. In contrast, head-fixed mice experience a constant noise intensity. Moreover, since head-fixed mice are unable to escape from the noise, they may lack the motivation to avoid it. This inescapable condition could influence dopaminergic transmission related to motivational processes, since motivation-related dopamine dynamics are known in the NAc.^42^^,^^43^ We assume that the results in our present study reflect primarily stimulus-driven dopaminergic responses to loud noise, as the motivational component of dopaminergic transmission would be suppressed under head-fixed conditions. Additionally, the sensitivity of the recording methods differs between the two studies. The previous study employed fast-scan cyclic voltammetry (FSCV) to measure dopaminergic dynamics, whereas we used fiber photometry with the genetically encoded dopamine sensor dLight1.3b. Recently, several studies reported that genetically encoded dopamine sensors provide higher sensitivity and temporal resolution at synaptic sites compared to FSCV, although FSCV offers superior detection of minimal dopamine concentrations.^44^^,^^45^^,^^46^ Therefore, the temporal profile of dopamine dynamics that we observed might not have been detected using the FSCV method. These factors would explain the discrepancy between the results in the previous study and our present results.

Adding to the dopamine decrease, we also confirmed that a steep transient increase of dopamine in the NAc at the onset of noise presentations occurred in several subjects. This steep increase was particularly prominent at the first noise burst, whereas responses to subsequent bursts were markedly reduced. Furthermore, the amplitude of the steep increase did not correlate with the intensities of the noise, distinct from the dopamine decrease that correlated with the noise intensities. These characteristics of the steep increase of dopamine suggest that this increase encodes the saliency of the noise as a novel stimulus. Previous studies have shown that dopamine activity in the NAc encodes not only valence but also the perceived saliency of incoming stimuli.^29^^,^^31^^,^^32^ Notably, the dopamine response to novel stimuli declines as their saliency fades.^32^ This temporal character is highly reminiscent of the steep dopamine increase we identified in this study, which was prominent only at the onset of the first noise burst and nearly absent in subsequent presentations. This similarity supports the idea that the steep dopamine increase in the NAc encodes the perceived saliency of the noise presentation.

By simultaneous recordings of the dopamine dynamics and neural activity in the NAc and TS, we revealed that neural activity increased during noise presentations in both NAc and TS, regardless of whether the direction of the dopamine signal changed. It is well known that dopamine generally stimulates D1-MSNs while suppressing D2-MSNs.^47^^,^^48^^,^^49^ These dopaminergic modulations on the excitability of MSNs are rapidly exerted on sub-second time scales.^50^^,^^51^ In the striatum and related regions, these two populations of neurons are spatially intermingled.^47^^,^^48^^,^^52^ Based on these well-established facts, we hypothesize that the opposite dopamine dynamics we observed in the NAc and TS lead to distinct, region-specific shifts in the activity balance between these two cell types. Specifically, in the NAc, the reduction in dopamine during noise presentations would lead to a disinhibition of D2-MSNs, resulting in their enhanced activity. This assumption is well-supported by previous studies reporting the involvement of NAc D2-MSNs activation in the anxiety-like behaviors and processing of negative valence.^53^^,^^54^^,^^55^^,^^56^^,^^57^ In contrast, in the TS, the dopamine increase would likely drive the preferential activation of D1-MSNs. This is consistent with the known role of TS D1-MSNs in promoting avoidance of the threat^27^ and similar D1-dependent defensive behaviors in the dorsal striatum.^58^ Taken together, our results and this proposed model suggest that salient noise may trigger a coordinated, yet opposing, modulation of D1-and D2-MSN pathways in the NAc and TS, which may contribute to the promotion of defensive behaviors.

The present study suggested that dopamine dynamics may encode the perceived saliency and negative valence of aversive noise. This finding is particularly significant given that passive defensive behaviors to salient auditory stimuli are known to shift flexibly through fear learning and emotional states.^14^^,^^15^^,^^59^^,^^60^^,^^61^^,^^62^ We assume that the dopamine dynamics we observed could be a critical neural substrate underlying these behavioral shifts. However, the precise circuits through which auditory information modulates the dopaminergic system remain largely unknown, although recent work has begun to identify direct auditory pathways to the VTA.^63^^,^^64^ Further future studies are required to delineate the specific auditory pathways that shape dopamine responses and determine how these dopamine signals causally contribute to the learning and state-dependent shifts in defensive behaviors.

### Limitations of the study

This study generally demonstrated, in both the NAc and TS, that as noise intensity increases, dopamine levels shift in the direction proposed by previous studies to reflect negative valence. However, the direct evidence that the dopamine dynamics drive defensive behaviors and the correlation between noise intensity and negative valence were not provided. These gaps could be addressed by neural manipulation using optogenetics or chemogenetics. Also, there were several technical limitations in the present study. The temporal resolution of fiber photometry is constrained by the kinetics of the genetically encoded biosensors; therefore, responses faster than the sensor kinetics may not be detectable in some cases. In addition, because we used a relatively large-diameter, high-NA fiber cannula, the recorded fluorescence likely reflects signals integrated over a broader tissue volume and may not originate exclusively from the region immediately adjacent to the fiber tip. These limitations could be addressed in future studies using electrophysiological recordings and/or mini-scope imaging.

## Resource availability

### Lead contact

Further information and requests for resources and reagents should be directed to and will be fulfilled by the Lead Contact, Ryo Yamamoto (ryamamot@kanazawa-med.ac.jp).

### Materials availability

This study did not generate new unique reagents.

### Data and code availability

• Data: Data reported in this paper will be shared by the lead contact upon request.
• Code: This study did not report original code.
• Other data: Any additional information required to reanalyze the data reported in this paper is available from the lead contact.

## Acknowledgments

This work was supported partly by 10.13039/501100001691KAKENHI grants JP25K00128 (to T.F.), JP24K12660 (to M.O.), JP25K12801 (to N.K.), JP24K10746 (to R.Y.), a grant from 10.13039/100019433Shibuya Science Culture and Sports Foundation (to M.O.), a financial support from Dr. Takuo Fujiwara (to M.O.), 10.13039/100012045Kawano Masanori Memorial Public Interest Incorporated Foundation for Promotion of Pediatrics (to T.F.), 10.13039/100008732The Uehara Memorial Foundation (to T.F.), The CASIO Foundation (to T.F.), 10.13039/100007428The Naito Foundation (to T.F.), 10.13039/501100004051Kato Memorial Bioscience Foundation (to T.F.), The Nakatomi Foundation (to T.F.), and 10.13039/501100004330Smoking Research Foundation (to T.F.).

## Author contributions

T.F. and R.Y. conceived and designed the research; T.F., M.O., N.K., and R.Y. performed experiments; T.F., R.Y., M.O., and N.K. interpreted the results of the experiments; T.F. and R.Y. prepared figures; T.F. and R.Y. drafted the manuscript; T.F. and R.Y. edited and revised the manuscript; all authors approved the final version of the manuscript.

## Declaration of interests

The authors declare no competing interests.

## STAR★Methods

### Key resources table

**Table:** 

REAGENT or RESOURCE	SOURCE	IDENTIFIER
**Bacterial and virus strains**		

AAV9-CAG-dLight1.3b	Addgene	# 125560-AAV9
AAV9-Syn-JRGECO1a	Addgene	# 100854-AAV9
AAV9-CAG-FLEX-GCaMP8f	Addgene	# 162382-AAV9

**Experimental models: Organisms/strains**		

C57BL/6JmsSlc	Shimizu Lab Supplies	Cat#C57BL/6JmsSlc
DAT-ires-Cre	Jackson Laboratory	JAX #006660

**Software and algorithms**		

MATLAB	Mathworks	RRID:SCR_001622
SMART	Panlab	RRID:SCR_002852
Facemap	Syeda et al.^66^	RRID:SCR_021513

### Experimental model and study participant details

#### Experimental models

C57BL/6JmsSlc (n = 65; 2-4 months old; both sexes) and DAT-ires-Cre (n =15; 2-4 months old; both sexes) were used in this study. C57BL/6JmsSlc mice were obtained from Shimizu Lab Supplies (Tokyo, Japan), and DAT-ires-Cre mice (stock #006660^65^) were purchased from The Jackson Laboratory. No significant influence of sex on the results was observed.

#### Maintenance/care

All mice were bred and maintained in our facility. Two to four animals were housed together in plexiglass cages (20 cm × 40 cm × 17 cm) under a 12 h light/dark cycle at 22-25°C. Food and water were available *ad libitum*. All experiments were conducted during the light cycle. No subject was used in more than one experiment.

#### Institutional permission

Experiments were performed in accordance with the guiding principles of the Physiological Society of Japan and were approved by the Animal Care Committee of Kanazawa Medical University (2024-15). All animal experiments complied with the ARRIVE guidelines and followed the National Institutes of Health guide for the care and use of Laboratory animals (NIH Publications No. 8023, revised 1978).

### Method details

#### Surgery

Subjects were anesthetized with isoflurane (1–2%) during surgical procedures. AAV9-CAG-dLight1.3b (100 nl; catalog # 125560-AAV9, Addgene, MA, USA) or AAV9-Syn-JRGECO1a (100 nl; catalog # 100854-AAV9, Addgene) was injected into the core of the NAc (in mm; AP: 1.2, ML: 1.4, from bregma; DV: -3.9 from the brain surface) or the TS (in mm; AP: -1.1, ML: 2.9, from bregma; DV: -2.8 from the brain surface) of C57BL/6J mice by using a microinjector (Nanoject II, Drummond Scientific Company, PA, USA). AAV9-CAG-FLEX-GCaMP8f (100 nl; catalog # 162382-AAV9, Addgene) was injected into the VTA (in mm; AP: -3.2, ML: 0.7, from bregma; DV: 4.4 from the brain surface) of DAT-Cre mice. After each viral injection, an optical fiber cannula (core diameter, 400 μm, NA = 0.5, RWD, Shenzhen, China) was implanted at 0.2 mm above the injection site. The optical fiber cannula and a small metal plate were attached to the skull using dental cement (Super Bond, Sun Medical Company, Shiga, Japan) for the head fixation and the *in vivo* fiber photometry experiments. After the surgery, subjects were singly housed for 2-3 weeks before the fiber photometry experiments.

#### Sound stimuli

All sound stimuli were created by MATLAB 2021a (MathWorks, Inc., Natick, MA, USA) with 48 kHz sampling frequency and 16-bit resolution. Sound stimuli were delivered via a sound card (UA-1010, Roland, Shizuoka, Japan) and an amplifier (M6100SA, Marantz, Kanagawa, Japan). An electrostatic speaker (ES1, TDT, Tucker Davis Technology, Alachua, FL, USA) was placed in front of the subject’s head at a distance of 5 cm. We used band-pass noise bursts (1-24 kHz, 500 ms on/off, 10 bursts, total 10 s) with 5 ms linear rise/fall times at four different sound intensities (45, 65, 85, and 105 dB), ramped noise (1-24 kHz, 500 ms on/off, 10 ramped noise, 495 ms rise, 5 ms fall, 85 dB at the peak), or damped noise (1-24 kHz, 500 ms on/off, 10 damped noise, 5 ms rise, 495 ms fall, 85 dB at the peak) Sound pressure level (SPL; referenced to 20 μPa) was calibrated using a condenser microphone (type 4156N, Aco, Tokyo, Japan).

#### Two-compartment noise avoidance test

A two-compartment box, composed of Zone A and Zone B, was used for this test (Figure 1A). The test consisted of three sessions. In session 1, the subject explored two compartments without any noise presentations for 10 min. In session 2 (10 min), the noise bursts (1-24 kHz, 500 ms on/off, 85 dB) were presented when the subject entered Zone A. The noise bursts terminated when the subject exited from Zone A. In session 3 (10 min), the compartment noise presented was reversed. When the subject entered Zone B, the noise bursts were presented. The noise bursts terminated when the subject exited from Zone B. The interval between each session was 15 seconds. The mice (n = 8) used in this experiment were not used in any other experiments. Subject behavior was recorded on video, and transitions between compartments were detected using SMART software (Panlab, Barcelona, Spain) based on the animal’s center of mass. Sound onset and offset were controlled based on SMART’s real-time detection.

#### Measurement of pupil size

Pupils of subjects were illuminated with an infrared LED and recorded by a CMOS camera (C310n, Logicool, Tokyo, Japan) at 30 fps. The pupil area was measured by using Facemap,^66^ an orofacial tracking software. The pupil area was normalized into z-scores based on the mean and the standard deviation of the 5 seconds before the onset of each trial for comparisons across different subjects. The noise bursts (10 sec, 500 ms on/off, 45 dB or 105 dB, 10 times) were presented for this experiment. The mice (n = 10) used in this experiment were not used in any other experiments.

#### *In vivo* fiber photometry configurations

The fiber photometry system was composed of a fluorescence mini-cube (iiFMC5, Doric Lenses, Quebec, Canada) with the appropriate dichroic mirror for excitation (465, 560 nm) and emission (525, 600 nm) light. The excitation light was controlled by an LED driver (Doric). Continuous excitation light (465 nm) was used for recording dLight1.3b and GCaMP8f signals. A 207 Hz sinusoidal excitation light (465 nm) and a 511 Hz sinusoidal excitation light (560 nm) were used for simultaneous recordings of dLight1.3b and jRGECO1a signals. The emission signals were sent to a fluorescence detector amplifier, and the signals were recorded by both a multifunction data acquisition device (USB-6346, National Instruments, Austin, TX, USA) at a 2 kHz sampling frequency and a Doric fiber photometry console at a 6 kHz sampling frequency. The resulting signals were down-sampled to 120 Hz and normalized into z-scores based on both the mean and the standard deviation of the 5 seconds before the onset of each trial. The z-score traces presented were smoothed with a 200-ms moving median.

#### Noise presentation experiments

Experiments were conducted in a sound-attenuated box. The noise bursts, ramped noise, or damped noise (described in the sound stimuli section) were presented for this experiment. Neural responses were recorded with the fiber photometry system. For the noise burst presentation experiments, subjects received 10-minute habituation on two consecutive days in the recording apparatus, then on Day 3, noise bursts (1-24 kHz, 500 ms on/off, 10 bursts, total 10 s) were delivered in a fixed order: 45 dB SPL ×10, then 65 ×10, 85 ×10, and 105 ×10. For the ramped/damped noise presentation experiments, subjects received 10-minute habituation on two consecutive days, then on Day 3 were presented with ramped (1-24 kHz, 500 ms on/off, 10 ramped noise, 495 ms rise, 5 ms fall, 85 dB at the peak) and damped (1-24 kHz, 500 ms on/off, 10 damped noise, 5 ms rise, 495 ms fall, 85 dB at the peak) noise. Ramped noise was presented 10 times, followed by 10 presentations of damped noise. Inter-stimulus intervals were randomly selected from 25, 30, or 35 seconds. No subject was used in more than one experiment, except, 7 subjects recorded from the TS underwent ramped and damped noise presentation experiments after noise burst presentation experiments. The maximum and minimum z-score values observed during noise presentation were defined as the “maximum z-score” and “minimum z-score”, respectively, representing the peak positive and negative deviations from baseline. Subjects were classified as “increase” type when the trial-averaged “maximum z-score” exceeded 2.58 at any of the tested intensities; the rest were classified as “decrease” type. The absolute differences between the z-score at noise onset and the values at these positive or negative peaks were defined as the “absolute z-score amplitudes”, reflecting the magnitude of signal change induced by the noise.

### Quantification and statistical analysis

Data are expressed as means ± standard error of the mean (S.E.M.) or robust S.E.M. A paired t-test followed by Bonferroni correction was used for multiple comparisons of the time spent in Zone A during the two-compartment noise avoidance test. Paired t-tests were also used to compare the mean pupil size, latency to the peak response, and minimum/maximum z-scores. The linear trends of minimum and maximum z-scores across the four noise intensities were assessed using subject-level cluster-robust (HC1) standard errors and a studentized wild cluster bootstrap-t (Mammen weights; B = 10000; two-sided; plus-one correction), with confidence intervals (CI) obtained by test inversion. For statistical testing, MATLAB 2021a was used. Statistical significance was set at p < 0.05.
