# Supplementary material for: Noise-evoked dopamine dynamics in the nucleus accumbens and tail of the striatum
Source: iScience. 2026 Apr 17;29(5):115789. doi: 10.1016/j.isci.2026.115789 (PMC13157026; doi:10.1016/j.isci.2026.115789)
Supplement: Document S1. Tables S1–S6 [file mmc1.pdf]

iScience, Volume *29*

Supplemental information

Noise-evoked dopamine dynamics

in the nucleus accumbens and tail of the striatum

Takafumi Furuyama, Munenori Ono, Nobuo Kato, and Ryo Yamamoto

Supplementary tables

Numbers for Fig.2 D1

Decrease type, minimum z-score (n = 9)

	45 dB	65 dB	85 dB	105 dB
First response	-0.30 ±0.06	-0.73 ±0.22	-1.32 ±0.20	-1.63 ±0.16
Average response	-0.18 ±0.08	-0.52 ±0.09	-1.14 ±0.10	-1.29 ±0.09

Numbers for Fig.2 E1

Decrease type, maximum z-score (n = 9)

	45 dB	65 dB	85 dB	105 dB
First response	0.95 ±0.10	0.86 ±0.25	0.78 ±0.20	0.56 ±0.19
Average response	0.76 ±0.09	0.80 ±0.07	0.65 ±0.08	0.89 ±0.16

Numbers for Fig.2 G1

Increase type, minimum z-score (n = 6)

	45 dB	65 dB	85 dB	105 dB
First response	-0.44 ±0.15	-0.60 ±0.10	-0.36 ±0.20	-1.30 ±0.33
Average response	-0.08 ±0.08	-0.24 ±0.09	-0.61 ±0.19	-0.99 ±0.27

Numbers for Fig.2 H1

Increase type, maximum z-score (n = 6)

	45 dB	65 dB	85 dB	105 dB
First response	0.89 ±0.27	3.76 ±1.76	3.60 ±0.54	3.78 ±0.30
Average response	0.17 ±0.08	0.73 ±0.30	0.79 ±0.14	1.17 ±0.13

Table S1. Average z-scores ±S.E.M are shown. Each table corresponds to the panels in Figure 2.

Numbers for Fig.3C

Decrease type, amplitudes of negative peaks (n = 9)

	1st	2nd	3rd	4th	5th
85 dB	1.28 ±0.25	1.37 ±0.14	1.19 ±0.24	1.19 ±0.24	1.15 ±0.28
105 dB	1.56 ±0.19	1.76 ±0.38	1.47 ±0.30	2.01 ±0.31	1.85 ±0.23

	6th	7th	8th	9th	10th
85 dB	1.29 ±0.28	1.42 ±0.24	1.14 ±0.22	1.37 ±0.28	1.05 ±0.23
105 dB	1.68 ±0.31	1.63 ±0.23	1.69 ±0.24	1.55 ±0.31	1.89 ±0.27

Decrease type, amplitudes of positive peaks (n = 9)

	1st	2nd	3rd	4th	5th
85 dB	0.48 ±0.25	0.17 ±0.07	0.20 ±0.09	0.29 ±0.09	0.37 ±0.11
105 dB	0.27 ±0.07	0.32 ±0.10	0.28 ±0.06	0.18 ±0.07	0.20 ±0.06

	6th	7th	8th	9th	10th
85 dB	0.29 ±0.10	0.20 ±0.11	0.26 ±0.12	0.20 ±0.06	0.34 ±0.12
105 dB	0.19 ±0.06	0.20 ±0.06	0.16 ±0.05	0.25 ±0.08	0.12 ±0.04

Numbers for Fig.3D

Increase type, amplitudes of negative peaks (n = 6)

	1st	2nd	3rd	4th	5th
85 dB	0.14 ±0.16	1.17 ±0.30	0.44 ±0.19	1.21 ±0.29	0.95 ±0.29
105 dB	0.38 ±0.21	1.07 ±0.15	1.31 ±0.52	2.11 ±0.41	1.83 ±0.47

	6th	7th	8th	9th	10th
85 dB	0.99 ±0.37	1.00 ±0.33	1.58 ±0.33	1.08 ±0.41	1.10 ±0.21
105 dB	1.63 ±0.30	1.77 ±0.29	2.20 ±0.41	1.53 ±0.20	2.14 ±0.40

Increase type, amplitudes of positive peaks (n = 6)

	1st	2nd	3rd	4th	5th
85 dB	3.59 ±0.59	0.36 ±0.10	0.45 ±0.15	0.47 ±0.13	0.26 ±0.12
105 dB	3.74 ±0.45	0.31 ±0.07	0.49 ±0.10	0.31 ±0.14	0.45 ±0.10

	6th	7th	8th	9th	10th
85 dB	0.30 ±0.08	0.29 ±0.11	0.20 ±0.07	0.39 ±0.15	0.33 ±0.11
105 dB	0.44 ±0.12	0.42 ±0.17	0.29 ±0.13	0.45 ±0.14	0.43 ±0.14

Table S2. Average z-scores ±S.E.M are shown. Each table corresponds to the panels in Figure 3.

Numbers for Fig.4 D1

Decrease type, minimum z-score (n = 12)

	45 dB	65 dB	85 dB	105 dB
First response	-0.54 ±0.10	-0.84 ±0.19	-0.69 ±0.18	-1.34 ±0.21
Average response	-0.44 ±0.05	-0.55 ±0.09	-0.82 ±0.13	1.40 ±0.14

Numbers for Fig.4 E1

Decrease type, maximum z-score (n = 12)

	45 dB	65 dB	85 dB	105 dB
First response	0.42 ±0.08	0.89 ±0.33	1.35 ±0.14	0.89 ±0.11
Average response	0.47 ±0.07	0.62 ±0.11	0.88 ±0.08	0.99 ±0.09

Numbers for Fig.4 G1

Increase type, minimum z-score (n = 3)

	45 dB	65 dB	85 dB	105 dB
First response	-0.48 ±0.21	-0.54 ±0.03	-0.49 ±0.28	-046 ±0.55
Average response	-0.45 ±0.08	-0.47 ±0.11	-0.57 ±0.13	-0.87 ±0.13

Numbers for Fig.4 H1

Increase type, maximum z-score (n = 3)

	45 dB	65 dB	85 dB	105 dB
First response	0.50 ±0.27	0.87 ±0.53	3.17 ±1.00	4.61 ±0.74
Average response	0.44 ±0.17	0.56 ±0.16	0.94 ±0.24	1.48 ±0.38

Table S3. Average z-scores ±S.E.M are shown. Each table corresponds to the panels in Figure 4.

Numbers forFig.5 D1

dLight1.3b, minimum z-score (n = 12)

	45 dB	65 dB	85 dB	105 dB
Average response	-0.30 ±0.13	-0.73 ±0.13	-1.12 ±0.17	1.32 ±0.16

Numbers forFig.5 E1

jRGECO1a, maximum z-score (n = 12)

	45 dB	65 dB	85 dB	105 dB
Average response	0.35 ±0.17	0.52 ±0.18	1.45 ±0.27	2.16 ±0.34

Table S4. Average z-scores ±S.E.M are shown. Each table corresponds to the panels in Figure 5.

Numbers forFig.6 E&F

minimum z-score (n = 7)

	45 dB	65 dB	85 dB	105 dB
First response	-0.63 ±0.14	0.02 ±0.15	-0.46 ±0.15	-0.47 ±0.17
Average response	-0.46 ±0.04	-0.31 ±0.04	-0.54 ±0.12	-0.25 ±0.10

Numbers forFig.6 G&H

maximum z-score (n = 7)

	45 dB	65 dB	85 dB	105 dB
First response	0.56 ±0.12	1.31 ±0.48	1.49 ±0.30	2.03 ±0.42
Average response	0.48 ±0.07	0.70 ±0.13	0.60 ±0.18	1.22 ±0.16

Table S5. Average z-scores ±S.E.M are shown. Each table corresponds to the panels in Figure 6.

Numbers for Fig.7 D1

dLight1.3b, maximum z-score (n = 5)

	45 dB	65 dB	85 dB	105 dB
Average response	1.11 ±0.20	1.99 ±0.29	2.61 ±0.41	3.06 ±0.18

Numbers for Fig.7 E1

jRGECO1a, maximum z-score (n = 5)

	45 dB	65 dB	85 dB	105 dB
Average response	0.53 ±0.19	1.13 ±0.35	1.43 ±0.28	1.84 ±0.30

Table S6. Average z-scores ±S.E.M are shown. Each table corresponds to the panels in Figure 7.
